# Compression Treatment of Aneurism

**Published:** 1871-12

**Authors:** 


THE COMPRESSION TREATMENT OF ANEURISM.—*The Lancet* gives in brief the scheme of treatment so successfully pursued by Dublin surgeons in this affection, as explained by Dr. Macnamara at a late meeting of the British Medical Association.

The patient having been brought into good general health, and being neither anæmic nor hyperæmic, an elastic compressor is applied to the upper part, say of the femoral artery, a second compressor being arranged some three or four inches lower. The upper instrument is then adjusted so as to control the artery, and just arrest the pulsation or the sac and no more. "A roster of intelligent students is now organized, and to them is intrusted the management of the case. Two are appointed to take charge of the patient for an hour, when they are relieved by two others, and so on during the day, whereby unwearied attention is secured dnring the period that pressure is kept up; and, as in Dublin, hospitals are visited at 9 A. M., the treatment commences about that hour and is kept up to 9 P. M., when all pressure is removed, and the patient is encouraged to take his night's rest undisturbed. Next morning the treatment is resumed, and so on until the cure is accomplished. Dr. Macnamara pointed out that thus every exertion is made to conduct the case with as little inconvenience as possible to the patient.

The occurrence of a sharp pain in the neighborhood of the knee is looked for as the harbinger of cure, since it depends upon the rapid enlargement of the colatteral circulation.

